# Stressed Out About *Plasmodium falciparum* Gametocytogenesis

**DOI:** 10.3389/fcimb.2021.790067

**Published:** 2021-12-02

**Authors:** Miho Usui, Kim C. Williamson

**Affiliations:** Department of Microbiology and Immunology, Uniformed Services University of the Health Sciences, Bethesda, MD, United States

**Keywords:** malaria, transmission, sexual differentiation, gametocyte, developmental regulation, bone marrow

## Abstract

Blocking malaria transmission is critical to malaria control programs but remains a major challenge especially in endemic regions with high levels of asymptomatic infections. New strategies targeting the transmissible sexual stages of the parasite, called gametocytes, are needed. This review focuses on *P. falciparum* gametocytogenesis *in vivo* and *in vitro*. Highlighting advances made elucidating genes required for gametocyte production and identifying key questions that remain unanswered such as the factors and regulatory mechanisms that contribute to gametocyte induction, and the mechanism of sequestration. Tools available to begin to address these issues are also described to facilitate advances in our understanding of this important stage of the life cycle.

## Introduction

Nearly half of the world’s population is still at risk of malaria (WHO, 2020). Through the 1980s and 1990s, the spread of *P. falciparum* parasites resistant to chloroquine, the mainstay of treatment since the 1950s, across Africa increased the annual malaria mortality rate above one million (Nuwaha, 2001). The introduction of artemisinin combination therapy in 2004 coupled with increased distribution of insecticide-treated bed nets and enhanced vector control efforts reduced the prevalence of malaria 27.9% and mortality declined 42.5% by 2017 (Weiss et al., 2019). Intense efforts to prevent, detect and treat the disease have allowed several countries to successfully eliminate malaria. Algeria and Argentina in 2019 and China and El Salvador in 2021 have been certified as malaria-free and globally, a total of 40 countries and territories have now been declared malaria-free (WHO, 2021). As more highly endemic countries move toward malaria elimination, comprehensive approaches to accurately define the human malaria reservoir and the impact of both symptomatic and asymptomatic infections are required to effectively interrupt transmission.

**Figure 1:**
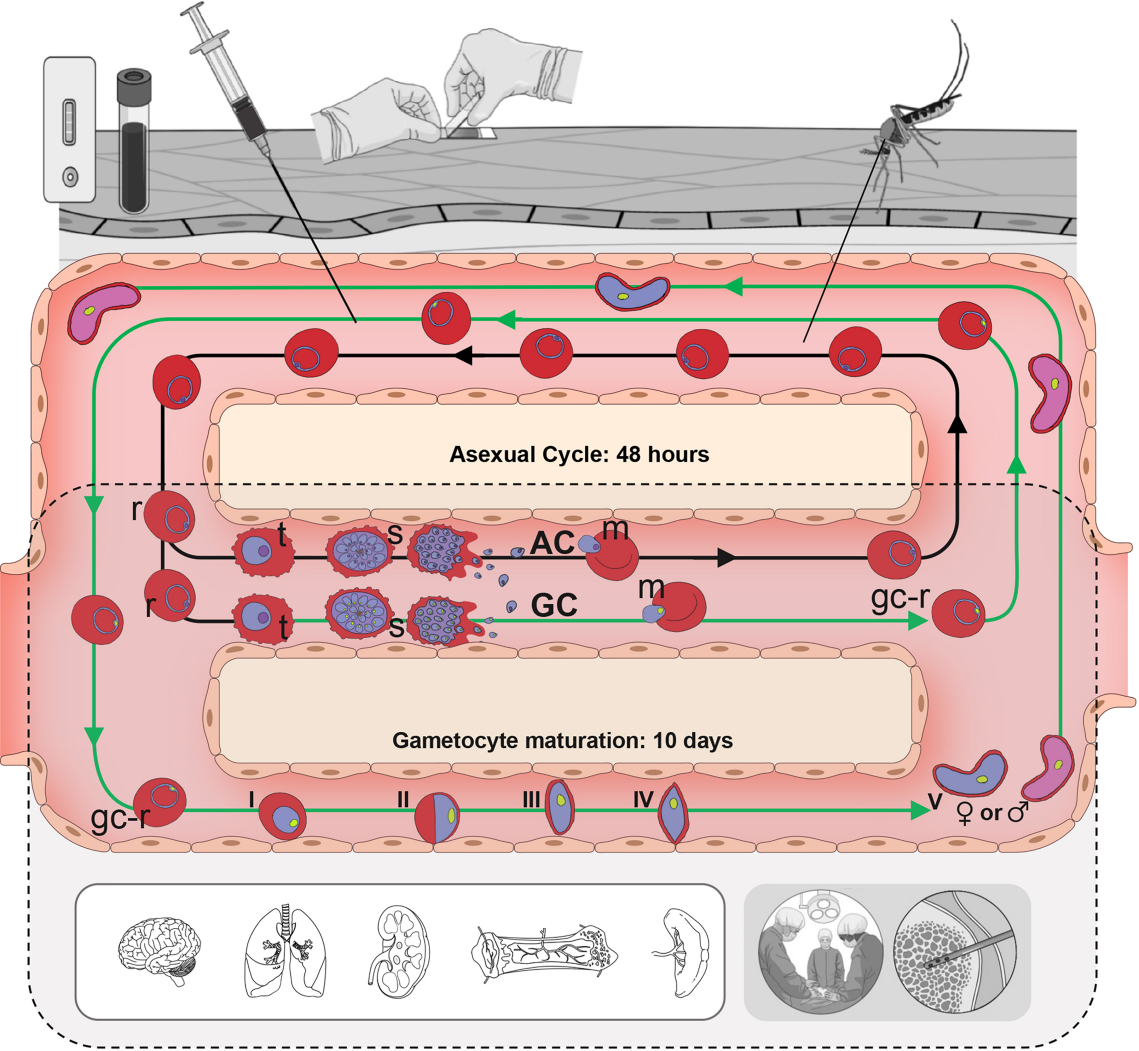
Human *Plasmodium falciparum* life cycle. In the bloodstream, asexually-committed (AC) merozoites (m, purple nucleus) invade RBCs and begin the asexual replication cycle (black arrows), passing through ring (r), trophozoite (t) and schizont (s) stages. During this development some parasites become gametocyte-committed (GC, green arrows) producing gametocyte-committed merozoites (green nucleus). Gc-merozoites invade RBCs and begin differentiating through 5 morphologically distinct stages (I-V) over the next 10 days to become mature stage V gametocytes. *P. falciparum* trophozoites, schizonts and immature stage I–IV gametocytes sequester and cannot be detected in peripheral blood samples (grey shading). Therefore, a blood sample obtained by a syringe, finger prick or mosquito bite only contains ac- and gc-rings and stage V gametocytes, while tissue samples are needed to collect all developmental stages.

Malaria infection begins when a *Plasmodium*-infected *Anopheles* mosquito introduces saliva containing parasites, called sporozoites, during a blood meal. Sporozoites travel through the bloodstream and invade liver cells where they replicate asexually producing 10,000-30,000 new merozoites that are released into the bloodstream when the liver cell ruptures. Merozoites invade red blood cells (RBCs) and either replicate asexually producing 16-32 new merozoites in 48 hours or differentiate into a single male or female gametocyte (**Figure 1**). The new merozoites are released by RBC rupture and the cycle repeats until it is controlled by the immune response or drug treatment. In contrast, the gametocytes produced during each cycle are terminally differentiated and die unless they are taken up in a blood meal by a mosquito. Once in the mosquito midgut, gametocytes are stimulated to undergo gametogenesis and fertilization resulting over the next 2 weeks in the production of tens of thousands of sporozoites that can be released with the saliva to initiate another infection.

This basic life cycle was described 121 years ago by Battista Grassi (Grassi, 1901) and at most steps a change in environment, either switching from host bloodstream to mosquito midgut or mosquito salivary gland to host liver, triggers the entire parasite population to begin a specific developmental program. The only exception to this for most species is the decision between asexual and sexual development during the intraerythrocytic cycle. Both stages develop in erythrocytes in the human bloodstream but have different outcomes. Asexual replication is required to maintain and increase parasite levels in the human host, while terminally differentiated gametocytes are required for transmission. If all the intraerythrocytic parasites commit to sexual differentiation at once, there would be no further increase in parasitemia and the infection would be over except for maturing gametocytes that survive for several days. The gametocyte circulation time would provide a small window for transmission *via* a mosquito to another person. Instead, the parasite has evolved so that only a subpopulation of parasites commits to sexual differentiation each cycle. This allows the host parasitemia to be maintained and provides a continuous supply of gametocytes that are available whenever a mosquito takes a blood meal.

## Gametocyte Development

**Figure 2:**
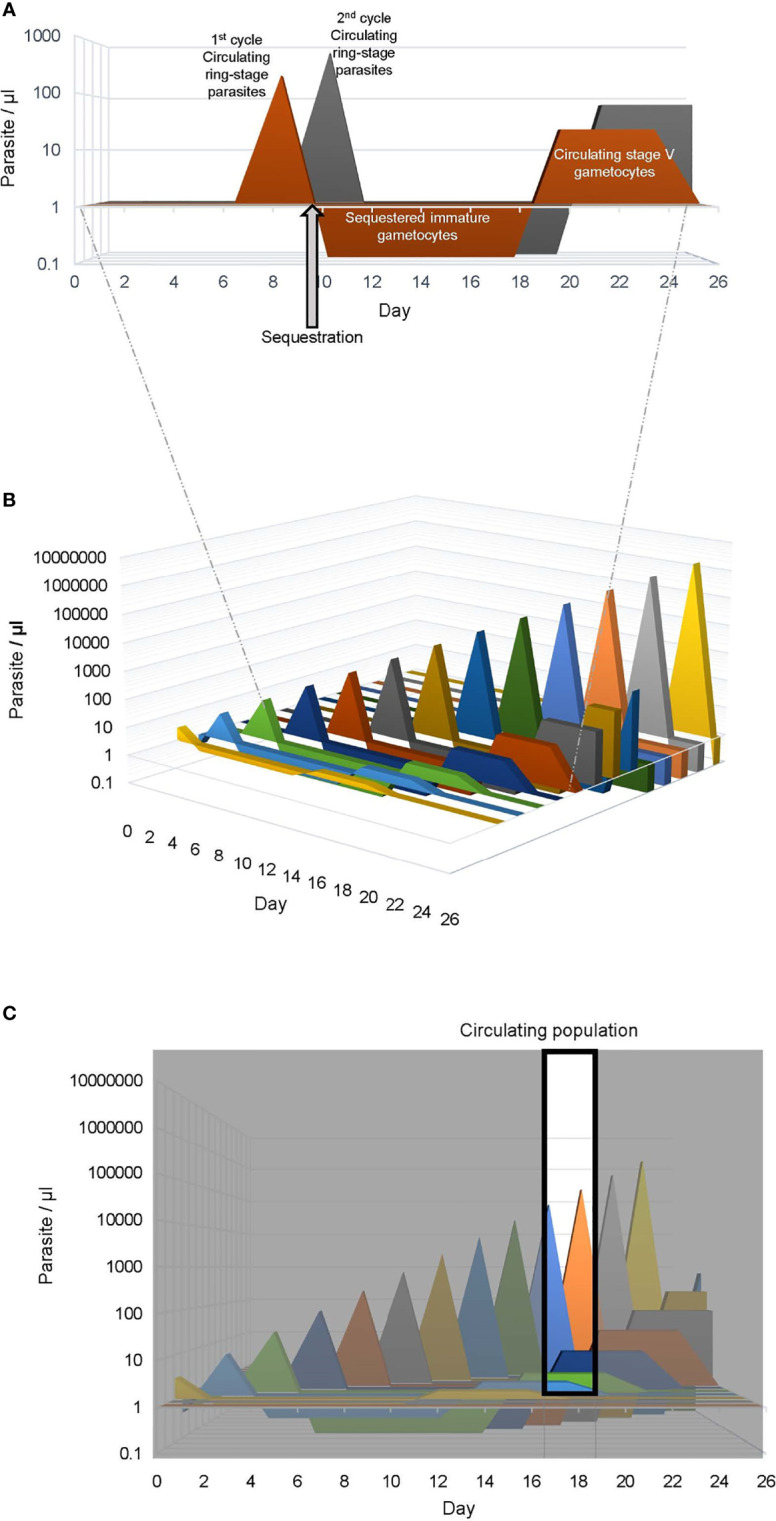
Human *Plasmodium falciparum* infection dynamics. Graphical representation of *P. falciparum* infection dynamics in human peripheral blood assuming a multiplication rate of three and a 10% sexual commitment rate. **(A)** The asexual ring stage and sexual stage parasites produced from 2 consecutive life cycles (1st, orange and 2nd, gray) is shown. The parasitemia of the circulating ring-stage and mature stage-V gametocytes is indicated as a positive value on the vertical axis, while the sequestered immature gametocyte parasitemia is indicated as a negative value. For clarity the sequestered mature asexual parasitemia is not included. **(B)** The graph is expanded to depict the asexual and sexual parasites produced in 13 sequential life cycles, mimicking a 26 day *P. falciparum* blood stage infection. **(C)** The window indicates the population of parasites collected in a blood sample obtained between day 17 and 19, which would include circulating ring stage parasites < 30 hours post RBC invasion from the 10th and 11th life cycles and mature stage V gametocytes (>10 days post RBC invasion) from the first 4 life cycles.

The time course of gametocyte production varies in different Plasmodium species. In *P. falciparum*, which is the focus of this review and responsible for the most virulent human malaria, gametocyte maturation within the RBC takes 10-12 days and has 5 distinct morphological stages (I-V) that can be observed in *in vitro* culture (Hawking et al., 1971). *In vivo* the time course is the same, but immature *P. falciparum* gametocytes (stage I-IV) are sequestered, primarily in the bone marrow and spleen (Thomson, 1914). The absence of immature gametocytes in peripheral blood samples complicates the evaluation of gametocytogenesis in the field. Once mature, stage V gametocytes are released back to circulation they survive a median of 6 days (Eichner et al., 2001). It is only during this time gametocytes can be picked up by a mosquito or in a blood sample for analysis using Giemsa-stained blood smears or reverse transcriptase-quantitative polymerase chain reaction (RT-qPCR). Gametocytes are not killed by current commonly used antimalarials and therefore, even after successful treatment, the long maturation and survival time of gametocytes allows continued transmission for over a week. At least one male and one female gametocyte must be taken up in a blood meal (~1 µl) for fertilization and further sporogonic development suggesting a theoretical limit of 2 gametocytes/µl or 0.00004% gametocytemia, which is below the sensitivity of a Giemsa stained smear. This means that the asexual parasitemia had to be at or above this parasitemia 10-12 days prior (**Figure 2**). Using the average sexual conversion rate observed in culture for *P. falciparum* strain NF54 of ~10% an asexual parasitemia of 0.0004% or 20 parasites/µl could produce enough mature gametocytes to spread the disease 10-12 days later. In malaria-endemic regions, asexual parasitemias in both asymptomatic and symptomatic carriers are often well above 20 parasites/µl suggesting that with a 10% conversion rate all these individuals could be important infectious reservoirs.

## The Infectious Reservoir

While young children < 5 years old report the highest rates of symptomatic *P. falciparum* cases, many people experience asymptomatic infection, including older children and adults (Doolan et al., 2009; Lindblade et al., 2013; Rodriguez-Barraquer et al., 2018). Although there is no standard definition of asymptomatic infection, it generally means a person with detectable circulating parasites but no fever or other acute symptoms (Lindblade et al., 2013). The increasing use of sensitive molecular diagnostics such as PCR revealed the high prevalence of asymptomatic infections and their potential as an infectious reservoir that could threaten malaria elimination in endemic areas (Lindblade et al., 2013; Goncalves et al., 2017). In contrast to individuals with acute symptomatic infections, those with asymptomatic infections do not seek treatment and are unlikely to be detected or treated (Nassir et al., 2005). Consequently, asymptomatic infections can be of longer duration and allow more time for gametocyte production (WWARN-Gametocyte-Study-Group, 2016). A recent multi-level logistic regression model that controlled for parasite density and mosquito abundance found that asymptomatic infections had 2.66-fold greater odds of malaria transmission to mosquitoes than symptomatic infections (Sumner et al., 2021). In a large study in Burkina Faso and Kenya, children, who harbor the highest parasite and gametocyte densities, are more infectious to mosquitoes than adults, but adults (> 15 yrs. old) have more exposure to mosquitoes, resulting in a greater contribution to falciparum infection in mosquitoes (Goncalves et al., 2017). Another concern is that asymptomatic individuals don’t know they are infected and can pass border screening and become a source of imported malaria under favorable conditions. This imported malaria is considered a serious challenge to malaria elimination (Lin et al., 2014; Domínguez García et al., 2019; Mischlinger et al., 2020) and has been the source of malaria resurgences in malaria-free countries (Nasir et al., 2020).

## Factors Associated With Gametocyte Carriage and *In Vivo* Gametocytogenesis

Although recent fieldwork has revealed the importance of asymptomatic infectious reservoirs, historically the factors associated with *P. falciparum* gametocyte carriage *in vivo* have been evaluated in symptomatic infections, which are much easier to monitor. A recent meta-analysis of clinical trials conducted on three continents, Asia, Africa, and South America, showed that, on average, gametocytes were detectable in blood smears in 12.1% of nearly 50,000 patients (WWARN-Gametocyte-Study-Group, 2016). Further analysis indicated that the prevalence of gametocytemia before treatment was negatively associated with age, parasitemia, fever (axillary temperature >37.5°C or reporting febrile symptoms), and hemoglobin concentration. This finding is consistent with previous reports of associations between stage V gametocyte prevalence and in low hematocrit (Drakeley et al., 1999; Stepniewska et al., 2008). A history of illness >2 days (Price et al., 1999; Sowunmi et al., 2004) has also been linked to stage V gametocyte carriage, but whether these factors are causal is difficult to determine from these studies. As mentioned before, stage V gametocytes collected in a blood sample began sexual differentiation 10-16 days before so it is possible that the low hemoglobin, and > 2-day history of fever could be associated with a longer infection (Garnham, 1931). Recent work monitoring immature and mature gametocytes in bone marrow aspirates and peripheral blood samples from anemic children also found a significant correlation between low hemoglobin levels, dyserythropoiesis, and mature gametocyte prevalence, but not immature gametocyte prevalence (Aguilar et al., 2014). These findings indicate that in this patient cohort low hemoglobin levels do not enhance sexual commitment, but rather suggest that gametocyte maturation in the bone marrow may interfere with erythrocyte production resulting in lower hemoglobin levels. Further work is needed in larger, more diverse populations to evaluate this more carefully.

To more directly evaluate clinical conditions closer to the time of initial sexual differentiation, blood samples from malaria patients were maintained in culture for 8 days in the presence of N-acetyl glucosamine (NAG) to block asexual growth and monitor gametocyte production (Usui et al., 2019). This approach revealed that 76% of the samples produced gametocytes, but the number of ring-stage parasites that differentiate sexually, referred to as the gametocyte conversion rate (GCR), varied from 0–78%. Gametocyte conversion correlated positively with the parasitemia of the initial ex vivo sample and a specific allele of a gene, *gdv1*, previously associated with gametocyte production *in vitro*. In contrast, fever and high lysophosphatidylcholine (LysoPC) levels had a negative influence. Neither patient hematocrit, age, nor leukocyte counts were associated with gametocyte commitment. The study also identified 3 molecular markers of sexual commitment in blood samples that can be used in place of the ex vivo assay facilitating future studies of larger cohorts to extend and refine the analysis of factors contributing to gametocyte commitment (Prajapati et al., 2020).

The *ex vivo* study described above was limited to subjects from a single site in Coastal Ghana and excluded HbS positive individuals. Therefore, neither human genetic variation nor environmental factors were evaluated although they have been associated with gametocyte production. It has been reported that people with HbAS, HbAC, and HbCC β-globin genotype have higher gametocyte densities and/or higher infection rates than those with the HbAA genotype (Robert et al., 1996; Gouagna et al., 2010). Lawaly et al. (Lawaly et al., 2010) also showed a significant human genetic contribution to gametocyte prevalence in individuals from family-based cohort studies from Senegal and Thailand. Intriguingly, this effect was apparent only in asymptomatic *P. falciparum* infections, not in symptomatic patients.

Climatic conditions that influence the growth and survival patterns of mosquito species with different vectorial capacities are additional factors that influence malaria transmission. In some areas, the prevalence of asexual parasites (Baird et al., 2002) and gametocytes (van der Kolk et al., 2003) have been reported to vary with season. Interestingly, in moderate and low transmission sites in western Kenya gametocyte levels are higher following the dry season than during the wet season when the prevalence of asexual parasites and clinical disease is high (Oduma et al., 2021). On the other hand, in coastal Ghana gametocyte prevalence increased during the dry season in an urban area, not a rural area (Ayanful-Torgby et al., 2018), demonstrating local variation and the need for large, long-term studies to understand the contributing factors. Higher expression of early gametocyte genes was also observed in regions with lower transmission levels when comparing sites in Kenya and Sudan (Rono et al., 2018) suggesting that parasites may adapt to the local transmission intensity. One hypothesis is that the parasite can sense the number of mosquito bites (Billingsley et al., 2005; Gadalla et al., 2016). However, there is no direct evidence for this association in humans or in rodent malaria models. In fact, in murine *P. chabaudi* and *P. vinckei* infections the gametocyte rate did not increase in response to mosquito probing (Shutler et al., 2005).

## *In Vivo* Gametocyte Localization and Development

Autopsy studies in the early 1900s identified immature gametocytes in the bone marrow and spleen, not other organs (Thomson, 1914), even though mature asexuals were found in other organs (Thomson, 1914; Garnham, 1931). The highest burdens of mature asexuals were seen in the brain and large intestines (Garnham, 1931), but this could vary depending on the pathology observed (Garnham, 1931). One hundred years later this finding was confirmed using molecular approaches to evaluate parasite burdens in autopsy samples from children that died of cerebral malaria. RT-qPCR analysis found the highest level of early gametocyte-specific transcripts in the bone marrow followed by the spleen (Joice et al., 2014). A higher level of immature gametocytes to mature gametocytes in the bone marrow versus peripheral blood has also been reported by Aguilar et al. (2014) and Smalley et al. (1981). Immunohistochemistry with a pan-gametocyte marker, anti-Pfs16 antibodies (Eksi et al., 2005), found the gametocyte/asexual parasite ratio significantly higher in bone marrow, even though total gametocyte levels were highest in brain and gut followed by the bone marrow, spleen, and heart (Joice et al., 2014). Subcellular localization studies in the bone marrow found equal numbers of gametocytes and asexuals in the parenchyma, while asexual parasites were more abundant intravascularly (Joice et al., 2014). A case study of a bone marrow sample from an adult malaria patient that had been treated with atovaquone, which cleared asexual parasites, also found immature gametocytes were more likely to be extravascular than mature gametocytes (Farfour et al., 2012). Similar localization studies have not been reported for the spleen which is a difficult biopsy but would be interesting.

The presence of extravascular parasites in the bone marrow raises several mechanistic questions. First, how did they get access? Second, does this environment enhance sexual differentiation? These questions will be addressed after a brief introduction to the bone marrow. The bone marrow is the primary site of hematopoiesis and produces 6 billion cells per kilogram of body weight per day (Davé and Koury, 2021) that enter the circulation by crossing the single layer of endothelial cells surrounding the vascular sinusoids. Egress occurs when blood cells mature to the point they no longer express the surface molecules needed to retain them in the bone marrow and the pressure gradient forces them through the endothelial layer into the vasculature (Waugh et al., 1984; Lichtman and Santillo, 1986; Beck et al., 2014). This egress model fits with studies demonstrating that immature gametocytes, stage II-IV are quite stiff and at physiologic pressures cannot pass through a matrix simulating the density of the spleen (Aingaran et al., 2012; Dearnley et al., 2012; Tibúrcio et al., 2012). Deformability increases as gametocytes transition to stage V gametocytes allowing them to move through the same matrix (Tibúrcio et al., 2012). Given the fenestrated endothelium of the spleen that allows passive trafficking of nonmotile RBC from the vasculature, it is possible that as gametocyte committed rings mature and stiffen they become trapped in the spleen until they again become deformable at stage V. However, this does not explain entry to the extravascular space of the bone marrow.

**Figure 3:**
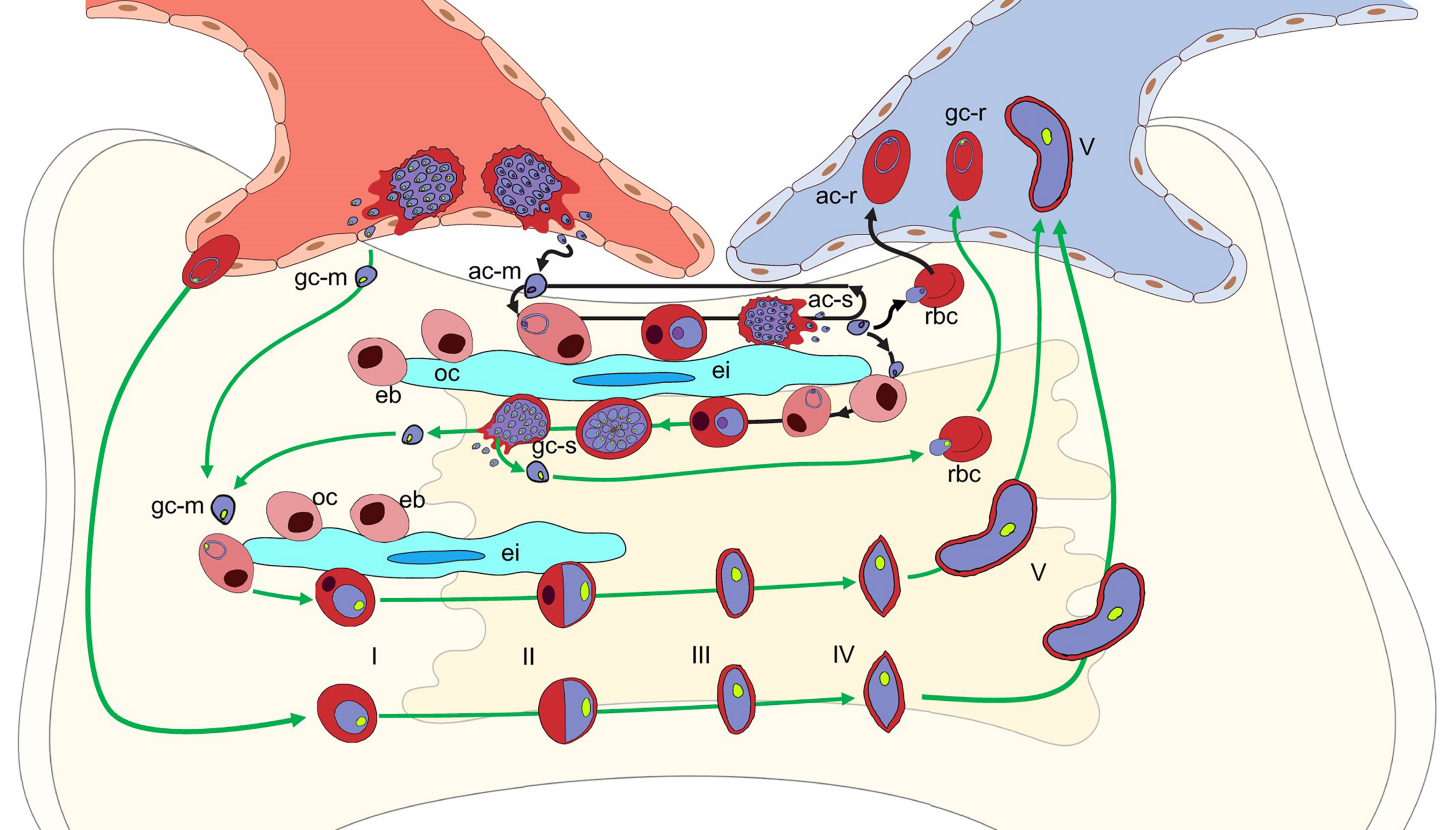
Potential *P. falciparum* developmental pathways in the bone marrow parenchyma. Schematic of potential developmental pathways for asexually-committed (ac-m, purple nucleus), gametocyte committed (gc-m, green nucleus) merozoites, or erythrocytes containing ring stage parasites. The mechanisms involved in the parasite’s exit from the circulation, across the endothelial cells into the bone marrow parenchyma remains unclear. Once in the parenchyma where erythroblastic islands (ei) support developing erythroblasts (eb), the ac-merozoite (black arrows) could invade a developing erythroblast after the orthochromatic stage (oc) and continue to develop into either an ac-schizont (ac-s) (main arrow) or, although not shown for clarity, a gc-schizont (gc-s). The ac-merozoite could also invade a newly formed RBC and be released back into the blood stream as a ring stage parasite (ac-r). Likewise, an ac-merozoite generated in the parenchyma could also have the same three fates and these are indicated in the figure. The gc-merozoite (green arrows) from the blood or produced in the bone marrow could invade a developing erythroblast after it transitions to the orthochromatic stage (oc) and continue to differentiate into a mature gametocyte (V) that is deformable enough to be released back into the blood stream. As with the as-merozoite, the gc-merozoite could also invade a newly formed RBC and be released back into the blood stream as a gc-ring stage parasite (gc-r). If erythrocytes containing gc-rings enter the bone marrow, they could also continue to develop through the stiff immature stages to a deformable mature stage V gametocytes that could be released to the circulation. Although not shown due to space constraints, if erythrocytes containing gc-rings enter it is likely that erythrocytes containing ac-rings could also enter and continue development into ac- or gc-schizonts.

Leukocytes and hematopoietic progenitor and stem cells expressing the appropriate adhesion molecules, CXCR4 and integrin α4β1 actively migrate into the bone marrow parenchyma in response to a CXCL12 gradient (Mazo et al., 2011), but the entry of RBCs has not been reported. One option for parasite access might be that a merozoite released by a schizont in the bone marrow sinusoids could bind to another cell as it enters the bone marrow, but this is speculation. Based on *in vitro* studies (Tamez et al., 2009; Neveu et al., 2020), once in the bone marrow parenchyma both asexually and sexually-committed merozoites could invade erythrocyte precursors developing there, starting from the late orthochromatic stage and complete development as the erythrocyte matures (**Figure 3**). If this is also the case *in vivo*, then after the infected cell matures it could be released into the circulation or the schizont could rupture in the bone marrow where the merozoites could reinvade another developing erythrocyte (**Figure 3**). It has been hypothesized that erythrcyte precursors preferentially select for sexual development (Peatey et al., 2013; Brancucci et al., 2017). However, there is strong ex vivo evidence that sexually-committed rings circulate for 13-20 hours before sequestration (Smalley et al., 1981; Usui et al., 2019; Prajapati et al., 2020). This has also been supported by transcriptomic data (Lemieux et al., 2009; Pelle et al., 2015; Prajapati et al., 2020).

Given the evidence for the circulation of sexually-committed rings, another alternative hypothesis for bone marrow access is the direct entry of RBCs infected with sexually or asexually-committed ring stage-parasites at least in the fatal malaria cases assessed by autopsy (Joice et al., 2014) and the anemic patients assessed by biopsy (Aguilar et al., 2014; Joice et al., 2014) (**Figure 3**). During malaria infection, there is an increase in granulocytes in the bone marrow, but a transient decrease in the numbers of lymphocytes, erythrocytes, and megakaryocytes (Villeval et al., 1990a; Villeval et al., 1990b). It is possible the inflammation induced by severe or chronic malaria infections may enhance the permeability of the endothelial cells of the sinusoids or the newly reported trans-cortical vessels (Aguilar et al., 2014; Grüneboom et al., 2019; Morikawa et al., 2020). Once in the parenchyma, the asexually or sexually-committed ring infected RBCs could be retained in the bone marrow during maturation by the expression of parasite encoded adhesion molecules on the surface of the infected RBC. Adhesion molecules, such as PfEMP1, localized to knob-like structures on the surface RBCs during asexual parasite maturation are known to be involved in sequestration to endothelial cells as well as syncytiotrophoblasts in the placenta (Leech et al., 1984; Rogers et al., 1996; Rogers et al., 2000). Sexually-committed parasites do not produce knob-like structures and have not been shown to adhere to endothelial cells but do produce a set of gametocyte-specific proteins that are predicted to be exported to the RBC (Eksi et al., 2005; Silvestrini et al., 2010; Tibúrcio et al., 2013). These proteins could be involved in adhesion at least until the gametocyte stiffens enough to prevent egress before maturation to stage V. There is also evidence for both asexually- and sexually-committed parasites binding to mesenchymal cells, but the specific parasite-produced proteins have not been identified (Messina et al., 2018). Inside the bone marrow, the parasites could continue sexual or asexual development and be released into the circulation as a stage V gametocyte, merozoite, or newly invaded developing RBC (**Figure 3**). The merozoites could also reinvade a developing erythrocyte in the bone marrow and begin asexual or sexual development (**Figure 3**).

Although as discussed above, there is no direct support that bone marrow or spleen environments enhance commitment to sexual differentiation, it is possible that their unique environments are optimal for gametocyte maturation. The relative distribution of fatty acids, including LysoPC, differ considerably from that in the serum (Brancucci et al., 2017). Studies in mice combining *in vivo* imaging with direct measurement of local concentration of oxygen (pO_2_) in the calvarial bone marrow suggest that the pO_2_ inside blood vessels sharply drops after vessels enter the bone marrow, an observation attributable to active O_2_ consumption by bone marrow cells (Spencer et al., 2014) and the association of hypoxia with hematopoietic stem cell quiescence (Hermitte et al., 2006; Eliasson and Jönsson, 2010). It is difficult to test this directly in humans, but the recently validated biomarkers for early sexually-committed ring-stage parasites can be used to assess the *in vivo* relationship between sexually-committed-rings and circulating gametocyte levels 10-11 days later. This information can be used to determine the association between gametocyte maturation *in vivo* and different clinical factors, including hematopoiesis, parasitemia, and fever as well as other factors recently found to influence gametocytogenesis *in vitro*.

## Molecular Mechanisms Contributing to Gametocytogenesis *In Vitro*

At the molecular level, *P. falciparum gametocyte* development 1 (*gdv1*, PF3D7_0935400) was the first gene found to play a major role in gametocyte production followed by the identification of ApiAP2 family transcription factor (*ap2-g*, PF3D7_1222600), which is regulated by GDV1 (Eksi et al., 2012; Filarsky et al., 2018; Usui et al., 2019). The loss of gametocyte production in lab strains is frequently associated with *Gdv1* deletions (Tiburcio et al., 2021), while GDV1 overexpression increases gametocyte production (Eksi et al., 2012; Filarsky et al., 2018). Field populations also exhibit high *gdv1* allelic divergence that has been suggested to vary with transmission patterns (Mobegi et al., 2014; Bungei et al., 2020). *Ap2-g* was also identified as the target of mutations in gametocyte-deficient parasite lines in both *P. falciparum* (Kafsack et al., 2014) and *P. berghei* (Sinha et al., 2014). During the asexual replication cycle *ap2-g* expression is silenced. Once *ap2-g* transcription is induced and AP2-G protein levels increase it enhances its own transcription (Kafsack et al., 2014; Sinha et al., 2014; Poran et al., 2017) and initiates gametocytogenesis through a cascade of gene expression that is tightly controlled through complex regulatory systems (Poran et al., 2017; Josling et al., 2018). A*p2-g* is epigenetically silenced by histone 3 lysine 9 trimethylation (H3K9Me3), which is stabilized by heterochromatin protein 1 (*hp1*, PF3D7_ 1220900) binding. Histone deacetylase 2 (*hda2*, PF3D7_1008000) which removes the acetyl group from H3K9 facilitating methylation has also been shown to affect gametocyte production (Brancucci et al., 2014; Coleman et al., 2014). This work has been described in detail in a number of recent reviews (Josling et al., 2018).

Although identified in *in vitro* culture adapted parasite lines, strains lacking *gdv1* and *ap2-g* have not been identified in the field. This difference is likely due to the need for gametocytes for transmission from person to person *via* a mosquito in the field, which is not required for propagation in culture. In fact, propagation in culture selects for gametocyte-deficient strains that produce more asexually-committed merozoites, instead of both asexually and sexually-committed merozoites. Despite the strong evidence that *gdv1* and *ap2-g* play major roles in gametocytogenesis, *gdv1+/ap2g+ P. falciparum* strains can have markedly different sexual differentiation rates (0 to 20%) (Brancucci et al., 2015; Poran et al., 2017) suggesting additional genetic factors play a role. There is also marked intra-strain variation in gametocytogenesis implying environmental modulation.

## *In Vitro* Gametocyte Production

In *P. falciparum*, several extracellular factors such as nutritional level, parasitemia, oxidative stress, cAMP, cGMP or lactate levels, and drug treatment have been reported to influence gametocyte conversion in *in vitro* culture (Buckling et al., 1999; Price et al., 1999; Sowunmi et al., 2006; Chaubey et al., 2014; West and Sullivan, 2020). However, using traditional *in vitro* culture methods the low yield of gametocytes and intra-strain variation, which can range from 0-3% gametocytemia, have made the reports difficult to replicate (See Buchholz et al., 2011, **Supplemental Data 1C**). Consequently, the specific stressors and signaling pathways that connect these environmental signals to parasite development have been elusive and instead lead to the general notion that stress induces gametocyte production (Dyer and Day, 2000; Baker, 2010; Josling et al., 2018).

Recent work by Brancucci et al. has advanced our understanding of the extracellular factors involved in regulating asexual growth and sexual differentiation. They identified a parasite line tagged with an early gametocyte reporter, Pf2004/164tdTom, that required serum-free media to make gametocytes. Using this line, they carefully fractionated serum components and found that the addition of LysoPC effectively reduced gametocyte production and increased asexual replication to the levels observed in media with serum (Brancucci et al., 2017). Tracking the metabolism of labeled LysoPC demonstrated that extracellular LysoPC is the parasite’s primary source for choline for the production of phosphatidylcholine (PC) (Pessi et al., 2004). PC is a major component of phospholipid membranes in all eukaryotic cells and is required for the production of the 16- 32 new merozoites every 48 hours. The mechanisms used to transport LysoPC from the plasma into the intraerythrocytic parasites remain unknown, but once inside the parasite the results demonstrate that the choline moiety is removed from LysoPC and used to synthesize PC *via* the Kennedy Pathway (Gibellini and Smith, 2010). When LysoPC is depleted from the media the parasite can survive for one asexual cycle by using phosphoethanolamine methyltransferase (PMT) to produce phosphocholine (P-choline) by trimethylation of parasite produced phosphoethanolamine (P-ethanolamine (Witola et al., 2008; Serrán-Aguilera et al., 2016; Brancucci et al., 2018). However, the number of new merozoites produced is reduced which results in fewer new ring-stage parasites (Brancucci et al., 2017). Interestingly, in the absence of LysoPC a higher proportion of the ring stage parasites produced are committed to sexual differentiation. Gametocyte commitment varies for different parasite strains, but even in the absence of LysoPC remains below 30% of the ring stage parasites. This indicates that some ring-stage parasites continue to replicate asexually in the absence of LysoPC, but the mechanism regulating this remains unknown. This differential response to the same media is consistent with environmental factors modulating, not triggering, sexual differentiation.

One proposed mechanism for the regulation of sexual conversion in the absence of LysoPC is lowering the intracellular levels of S-adenosylmethionine (SAM). SAM is the substrate PMT uses to trimethylate P-ethanolamine to produce P-choline for the synthesis of PC. SAM is also used to methylate histones. Since sexual conversion is repressed by histone trimethylation mediated silencing of *ap2-g*, it has been suggested that the increased use of SAM to trimethylate P-ethanolamine could reduce histone methylation thereby enhancing *ap2-g* transcription and sexual conversion (Llinás, 2017). This is an intriguing hypothesis and awaits further experimental testing. The feedback pathway that regulates the upregulation of PfPMT in response to low choline levels is also unknown.

## Discussion

The dual role of LysoPC depletion in reducing asexual growth and enhancing gametocyte production complicates dissecting the specific mechanisms involved. It also highlights the need for careful assessment of both asexual growth and gametocyte production. Both are interrelated because during each asexual cycle both asexually and sexually committed-schizonts, -merozoites, and -newly invaded rings are formed. If a treatment affects asexual growth so that the treated culture forms fewer new parasites, the number of gametocytes produced would also be lower. It is relatively easy to control for this by using gametocyte conversion rate (GCR) instead of just gametocytemia to monitor gametocyte conversion (**Table 1**). The GCR is calculated by dividing the stage II gametocytemia on day (D) 4 or stage III gametocytemia on D6 by the total ring/trophozoite stage parasites on D0-D1 before blocking asexual replication using NAG (**Table 1**, column H). Asexual replication should be blocked using NAG because subsequent asexual cycles also produce sexually-committed gametocytes, which can complicate quantification. This continuous gametocyte production also means that gametocytes generated during earlier cycles are also present and need to be excluded during quantification. This is a particular problem if the culture has been maintained at a high parasitemia for several cycles and can result in a high gametocyte background. One way to control for this is to only count stage II gametocytes on D4 or stage III on D6.

**Table 1:** Gametocyte conversion calculation table.

	B	C	D	E	F	G	H	I	J
1	Group	Set up	Initial parasitemia cycle			G-cyte	G-cyte conversion		
2		Parasitemia (%)	Next cycle (D0) parasitemia (%)	Multiplication rate (MR)	Control MR	D4 or D6 g'cytemia (%)	Original GCR (%)	RBC-rupture-corrected GCR (%)	Control MR-corrected GCR^#^ (%)
3^*^				=D/C	=E4		=(G/D)*100	=((G/(100-D)*100)/D)*100	=((G/(100-D))*100)/(C*F)*100
4	Control	2.00	8.68	4.34	4.34	0.87	10.02	10.98	10.98
5	Pseudo-induction	2.00	5.19	2.59	4.34	0.90	17.34	18.29	10.94
6	Induction	2.00	8.19	4.09	4.34	1.88	22.95	25.00	23.59

*Row 3: The formulas provided can be used to calculate the different gametocyte conversion rates (GCR) for different experimental conditions from the set up (column C) and D0 asexual cycle parasitemias (column D), the control multiplication rate (column F), and the gametocytemia (column G).

^#^Row 5: If the control-corrected GCR (column J) is the same as the RBC rupture-corrected GCR for the Control group (column I & J, row 5) then additional experiments are needed to confirm that the production of asexually-committed, D0 rings was not preferentially affected by the induction conditions.

One major caveat to the use of the GCR for *in vitro* cultures is the observation that asexually replicating parasites die or “crash” (Saliba and Jacobs-Lorena, 2012; Delves et al., 2016) when parasitemia rises much above 5-6% while developing gametocytes are more resistant and can continue to mature. The differential resistance of asexually replicating and sexually differentiating parasites to crashing was the original strategy used to isolate gametocytes. The asexual parasitemia was allowed to increase in the culture until they all died, leaving a mixed population of gametocytes at different developmental stages. The specific factors that contribute to parasite death have not been identified, but Chou et al. (2018) showed that death began before schizogony. However, whether this differs for asexually and sexually-committed schizonts has not been tested. If asexually-committed schizonts are preferentially affected then the total number of rings would decrease, while the number of sexually-committed rings remains the same, thereby increasing the GCR in the absence of an actual increase in sexual conversion.

Asexual and gametocyte stages are also known to differ in their sensitivity to drugs that target DNA replication (Buchholz et al., 2011; Collins et al., 2019) and sorbitol lysis (Saul et al., 1990). Sorbitol resistance suggests a difference in permeability, perhaps due to the lack of the parasite-specific anion channel (PSAC), but this has not been definitely demonstrated (Saul et al., 1990; Nguitragool et al., 2011). This lower permeability could reduce the uptake and response of gametocytes to compounds in the media and again lead to a preferential decrease in asexually-committed, not sexually-committed parasites leading to an apparent increase in GCR. One option to control for this is to monitor the number of merozoites produced and the asexual multiplication rate. The asexual multiplication rate is the increase in parasitemia from one asexual generation to the next. Both the total number of merozoites produced and their invasion rate contribute to the multiplication rate. If conditions alter the multiplication rate, as observed when the culture crashes, then further analysis is needed to differentiate between alterations in asexual and/or sexual differentiation (**Table 1**). Another alternative is to quantify the total number of schizonts, newly invaded rings, and gametocytes produced instead of using parasitemia and gametocytemia. However, this remains technically challenging due to errors introduced during the large dilution needed to count individual RBCs. The lack of markers for sexually-committed schizonts and merozoites also complicates the analysis.

To facilitate tracking the transition from schizonts to gametocytes and determine whether treatment alters parasite growth and/or sexual commitment, a spreadsheet was developed (**Table 1**). The spreadsheet uses initial parasitemia for each flask, the ring/trophozoite parasitemia of the next asexual cycle (D0) to calculate the multiplication rate and the D0 parasitemia and stage II gametocytemia on D4 or stage III gametocytemia on D6 to calculate the GCR. The observed gametocytemia is then corrected for the loss of infected RBC during schizont rupture (RBC-corrected GCR, **Table 1**, column I), which can be substantial at high starting parasitemias. If the test culture has a different multiplication rate than the control culture, then the GCR for the test culture can also be recalculated using the number of rings that would have been produced with the control multiplication rate (MR). If this control MR-GCR (**Table 1**, column J) now equals the GCR of the control group, further analysis is required to confirm whether the original GCR was due to an increase in sexual conversion or a selective decrease in asexually-committed parasites. This detailed analysis of both asexual and gametocyte production should facilitate the identification of the sources of variability in gametocyte production, allowing more accurate and reproducible detection of induction conditions and ultimately replacing “stress” with actual regulatory mechanisms.

## Summary

Key genes, *ap2-g*, *gdv1* and *hp1*, involved in the initiation of *P. falciparum* sexual differentiation have been identified, but the mechanisms regulating induction in just a subpopulation of developing schizonts remains a mystery. Inter and intra-strain differences in sexual commitment in wild type (*ap2-g+/gdv1+/hp1+*) parasites suggest additional genetic and environmental factors play a role. Sensitive biomarkers for sexually-committed ring-stage parasites coupled with careful assessment of parasite growth and differentiation *in vitro* provide tools to extend the analysis to a range of environmental factors. Of particular interest are the roles of LysoPC and bone marrow and spleen sequestration, as well as quantifying gametocyte-committed rings and their maturation to gametocytes in asymptomatic and symptomatic individuals. Understanding the factors contributing to the infectious reservoir will be important to design control strategies for malaria elimination.

## Author Contributions

MU and KW both conceived of, researched, and wrote the manuscript. All authors contributed to the article and approved the submitted version.

## Funding

This work was supported by Public Health Service grants AI147627 and AI 114761 from the National Institute of Allergy and Infectious Diseases (NIAID).

## Author Disclaimer

The opinions and assertions expressed herein are those of the author(s) and do not necessarily reflect the official policy or position of the Uniformed Services University or the Department of Defense.

## Conflict of Interest

The authors declare that the research was conducted in the absence of any commercial or financial relationships that could be construed as a potential conflict of interest.

## Publisher’s Note

All claims expressed in this article are solely those of the authors and do not necessarily represent those of their affiliated organizations, or those of the publisher, the editors and the reviewers. Any product that may be evaluated in this article, or claim that may be made by its manufacturer, is not guaranteed or endorsed by the publisher.
